# Editorial: TNFRSF agonists: mode of action and therapeutic opportunities

**DOI:** 10.3389/fimmu.2023.1328100

**Published:** 2023-11-07

**Authors:** Nataša Obermajer, Adam Zwolak, Harald Wajant

**Affiliations:** ^1^ Oncology R&D, Johnson and Johnson Innovative Medicine, Beerse, Belgium; ^2^ Therapeutics Discovery, Johnson and Johnson Innovative Medicine, Spring House, PA, United States; ^3^ Division of Molecular Internal Medicine, Department of Internal Medicine II, University Hospital Würzburg, Würzburg, Germany

**Keywords:** agonism, antibody design, co-stimulation, Fcgamma receptor, immunostimulation, immunotherapy, signaling/signaling pathways, TNFRSF

The receptors of the tumor necrosis factor (TNF) receptor superfamily (TNFRSF) fulfill crucial and manifold immunomodulatory functions and are involved in maintenance of tissue homeostasis and development. TNFRSF receptors (TNFRs) exhibit specific, though partially overlapping expression profiles, and elicit functional effects on the vast majority of immune cells at different stages of an immune response. TNFRs also crucially contribute to the communication between immune cells and immune cells with other cell types. The repertoire of immunomodulatory functions of TNFRs spans both adaptive as well as innate immunity. For example, TNFRs co-stimulate T-cells, engage antigen presenting cells (APCs) and regulate B-cell maturation. On the other hand, they contribute to tumor surveillance and are also involved in tissue regeneration and development.

In line with the diverse functions of TNFRs, inhibition as well as activation of these receptors (or their ligands) has considerable potential for the treatment of a variety of immune pathologies, cancers, and infectious diseases. Agents targeting TNFR1 and TNFR2, RANK and BAFF-R pathways have long been approved for the therapy of autoimmune diseases (TNF blockers) (1), osteoporosis and giant cell tumors of bone and bone metastases of solid tumors (anti-RANKL antibody) (2) and systemic lupus erythematosus (anti-BAFF, TACI-Fc) (3, 4). For more than the past two decades there have also been tremendous preclinical and clinical efforts to bring TNFR agonists into the clinic, especially as cancer therapeutics. However, to date these efforts have resulted in only a single niche approval of recombinant TNF for the treatment of soft tissue sarcomas in isolate limb perfusion (5). The lack of translational success of TNFR agonists can be largely attributed to the challenging design of potent agonists due to the special mode of TNFR activation, and dose limiting toxicities arising from systemic TNFR activation. The later can be further exacerbated by FcγR binding in the case of TNFR-specific agonistic antibodies. Therapeutic targeting of TNFRs with agonists remains in a nascent stage. However, the improved molecular understanding of TNFR activation achieved in recent years prompted new concepts guiding the design of TNFR agonists with potent and/or conditional agonism. Ongoing and forthcoming clinical trials will be essential to validate the efficacy and safety of these novel agonists.

This Research Topic, compiling 7 review/perspective articles and 4 original studies, provides an overview of the molecular mode of action, as well as the clinical development and possible applications of TNFR agonists. The reviews deliver a comprehensive summary of our current understanding of the mechanisms of TNFR activation, and further outline how these learnings have shaped and changed the development of TNFR agonists in recent years, spotlighting 4-1BB agonists as an example. The original articles build on this premise and, with additional examples of several therapeutically relevant TNFRs, illustrate how current knowledge of TNFR agonism can guide the rational design of novel TNFR-activating therapeutics.

Vanamee and Faustman review the current state of our understanding of TNFR signaling mechanisms and present a uniform model of TNFR activation that can accommodate all members of the TNFRSF. The model highlights the importance of pre-formed hexagonal honeycomb-like TNFR clusters in instructing the recruitment of a likewise hexagonal assembled honeycomb lattice of downstream components to trigger the intracellular signaling pathways culminating in the induction of cell death or activation of the NFκB pathway upon engagement of the death domain-containing and TRAF-interacting receptors of the TNFRSF, respectively. Based on this model, Faustman and Vanamee furthermore discuss the possible underlying mechanism of action of antagonistic and agonistic anti-TNFR antibodies, with a goal to aid the development of better therapeutics.

Fromm et al. continue on the realm of oligomerization being the decisive prerequisite for TNFR activation and further discuss the ability of different types of synthetic agonists to facilitate higher-order clustering of TNFRs. The focus of their review is to synthesize how the available human clinical data reflect the underlying mechanisms of synthetic agonist compounds that have been evaluated in the clinic. Fromm et al. present examples of frequently observed ‘bell-shaped’ dose response effects in patients and highlight the variables to consider in selecting an optimal dose for TNFR agonists and common themes across different TNFR agonist modalities that should be considered in advancing future agents to the clinic.

In their review, Dadas et al. advance the discussion by describing the biomedical rationale, efficacy, and limitations of the currently available agents delivering co-stimulatory TNFR agonism for cancer immunotherapy. They propose considerations for the development of next generation immunostimulatory agents to overcome challenges in translating pre-clinical successes into the clinic. Dadas et al. provide an overview of the co-stimulatory TNFR targeting agents in clinical trials and list examples of preclinical and clinical studies of targeting T cell co-stimulatory TNFRs 4-1BB, CD27, GITR, OX40 and TNFR2 and the APC stimulatory CD40 receptor. Building on a summary of the recent approaches in targeting these TNFRs, they supplement with a discussion of potential modifications to achieve curative clinical immune responses while avoiding toxicity.

Apart from addressing common features of TNFR activation and the development of TNFR agonists in a rather general way, the next three reviews by Salek-Ardakani et al., Liu and Luo, and Chen et al. focus specifically on 4-1BB and TNFR2 to comprehensively cover therapeutic targeting of these two receptors.

Salek-Ardakani et al. begin by describing the structure of 4-1BB and the mechanisms of action of 4-1BBL and antibody-based agonists, including structural superposition of several agonist modalities targeting 4-1BB. The authors then summarize some of the major clinical efforts agonizing 4-1BB to date in immuno-oncology. In particularly, they outline the knowledge gained from the early studies with the two prominent 4-1BB therapeutics urelumab and utomilumab, provide a perspective on strategies that are being attempted to generate greater specificity in targeting and biological activity, and highlight opportunities in other clinical arenas such as viral vaccines and autoimmunity that have yet to be pursued.

In a related review on 4-1BB, Liu and Luo present 4-1BB biology in the context of anti-4-1BB agonist drug discovery. Comparing anti-4-1BB antibodies urelumab, utomilumab, and ADG106, Liu and Luo discuss in detail the relevance of the binding epitope and ligand-blocking properties in inducing 4-1BB clustering and signaling activation, the role of FcγR binding and antibody isotype for agonistic activity and regulatory T cell depletion, and the preferential reduced affinity and higher dissociation rate for agonism. They delve into strategies for conditional activation of 4-1BB to improve the therapeutic index by localizing the agonistic activity, and further describe the vast potential of combinatorial approaches either as multi-specific antibodies, in combination with cancer vaccines or T cell engaging antibodies.

Chen et al. provide a perspective on the therapeutic potential of TNFR2 agonists and their use to stimulate immunosuppressive regulatory T cells (T_reg_ cells) and myeloid derived immunosuppressive cells (MDSCs) for the treatment of autoimmune diseases, and also their potential use as co-stimulators of cytotoxic T lymphocytes (CTLs) and activators of NK cells in tumor therapy. An overview of TNFR2 agonists is intervened with most relevant factors that may determine their therapeutic outcome. The intricate bi-phasic effects of TNF-TNFR2 signaling, dual role of TNFR2 on T_reg_ cells and effector CD8 and NK cells and tissue-specificity of responses are discussed to explain the complicated nature of TNFR2 agonist responses.

In their article, Vredevoogd and Peeper indirectly address the development of TNFR agonists, by providing examples of proof-of-concept analyses of mechanisms of TNF resistance. By exploiting heterogeneity of DepMap dependency database, Vredevoogd and Peeper present an analytical approach for discovery of novel immune sensitivity modifiers. Comparing gene perturbation effects between cell lines that are positive vs negative for expression of TNF and TRAIL signaling intermediates, respectively, they validate previously described data and identify novel immune sensitivity modifiers. They further probe the fidelity of such an approach by comparing the drug sensitivity profile of these specific tumor cell lines in the DepMap.

The series of the above 7 review/perspective articles included in this Research Topic, is accompanied by 4 original research manuscripts, complementing the discussion on critical aspects of TNFRSF structure-function and receptor clustering with pre-clinical research data. The research papers focus on the requirement and role of FcγR binding in anti-tumor immunity and provide possible alternatives to achieve desired TNFRs agonism.

Melo et al. created a novel immune co-stimulatory CD27xEGFR IgG1 bispecific antibody lacking effector function and present its *in vitro* characterization in their research article. They describe selective and simultaneous binding of this tetravalent CD27xEGFR bsAb to both targets (CD27 and EGFR), T cell co-stimulation in co-cultures with a range of EGFR^+^ cell lines, and anti-cancer activity - both by co-stimulation of T cells at the sites of EGFR expression as well as by directly blocking EGFR on cancer cells. Melo et al. argue the unique features of CD27xEGFR and offer a compelling rationale for its further exploration in preclinical and clinical settings as a promising immunotherapeutic agent for EGFR^+^ tumors.

Dadas et al. performed pre-clinical side-by-side comparisons of soluble variants of CD70 (either trimeric (t) or dimer-of-trimers (dt)) to an agonist anti-CD27 antibody. Whereas tCD70 failed to co-stimulate CD8^+^ T cells, both dtCD70-Fc and an agonist anti-CD27 antibody could enhance T cell proliferation *in vitro*. When evaluating the dependence on FcγR binding, the activity of anti-CD27 antibody and dtCD70-Fc in FcγR-deficient mice remained active. Nevertheless, although a substantial part of the stimulatory activity of dtCD70-Fc was retained in the absence of FcγR interaction, FcγR binding of dtCD70-Fc was required for maximal induction of a CD8^+^ T cell response *in vivo*. Their data reveal that TNFSF ligands can be generated with a tunable activity profile and suggest that this class of immune agonists could have broad applications in immunotherapy.

In their comprehensive *in vitro* assessment of the individual contribution of different human FcγR classes on the agonistic activity of antibodies targeting 4-1BB (urelumab and utomilumab) and CD27 (varlilumab), Leitner et al. used a T cell reporter system to show that urelumab could induce 4-1BB signaling without FcγR cross-linking, but also that the presence of the FcγRs CD32a/b and CD64 augmented intrinsic agonistic activity of this antibody. However, utomilumab and varlilumab exerted agonistic function only when crosslinked (utomilumab via CD32A/B and varlilumab via any FcγR). In addition, they analyzed the capacity of these TNFR agonistic antibodies to augment PBMC activation. While the 4-1BB agonists induced T cell activation comparably well as a CD3 antibody alone, the capability of the CD27 agonist varlilumab to augment T cell responses in primary human PBMCs was counteracted by its FcγR-mediated cytotoxic effects. The data by Leitner et al. highlight the importance to account for the FcγR-mediated effects, such as ADCC and AICD, which critically impact the activity of antibody-based co-stimulatory TNFR agonists.

Zaitseva et al. generated various oligovalent variants of the fibroblast growth factor (FGF)-inducible 14 (Fn14)-specific antibody 18D1 and compared their agonism with that of soluble and membrane TWEAK (sTWEAK and memTWEAK), the natural ligands of Fn14 engaging different patterns of Fn14-associated signaling pathways. In their research article, Zaitseva et al. present that the number and type of the Fn14 binding domains within an oligovalent 18D1 construct determine whether sTWEAK- or memTWEAK-like activity is mimicked, and hypothesize that qualitatively different TNFR agonism with preference for specific TNFR-associated signaling pathways can be achieved by modifying the antibody design. Further, using one of their intrinsically agonistic 18D1 variants, Zaitseva et al. provide evidence that Fn14 activation *per se* can elicit anti-tumoral response and argue that apart from blocking Fn14 pro-tumoral activity or targeting it as a tumor associated antigen (6), inducing Fn14 agonism may serve as an alternative targeting approach for tumor therapy.

In conclusion, this Research Topic provides comprehensive overview of the TNFRs, focusing on the mechanisms of their activation, and their potential as therapeutic targets. Combining the data from pre-clinial research with the findings from clinical studies, the articles address the imminent challenges of TNFR activation related to clustering-enabled signal transduction and necessity for conditional agonism to avoid systemic activation.

## Author contributions

NO: Conceptualization, Writing – original draft, Writing – review & editing. AZ: Writing – review & editing. HW: Conceptualization, Writing – original draft, Writing – review & editing.
